# Growing role of concrete in sand and climate crises

**DOI:** 10.1016/j.isci.2023.106782

**Published:** 2023-04-29

**Authors:** Takuma Watari, Zhi Cao, André Cabrera Serrenho, Jonathan Cullen

**Affiliations:** 1 Material Cycles Division, National Institute for Environmental Studies, 16-2, Onogawa, Tsukuba, Ibaraki 305-8506, Japan; 2 Department of Engineering, University of Cambridge, Trumpington Street, Cambridge CB2 1PZ, UK; 3 Energy and Materials in Infrastructure and Buildings (EMIB), University of Antwerp, Antwerp, Belgium

**Keywords:** Environmental science, Global change, Civil engineering

## Abstract

Concrete production poses multiple sustainability challenges, including resource over-exploitation and climate change. Here we show that growing global demand for buildings and infrastructure over the past three decades has quadrupled concrete production, reaching ∼26 Gt/year in 2020. As a result, annual requirements for virgin concrete aggregates (∼20 Gt/year) exceeded the extraction of all fossil fuels (∼15 Gt/year), exacerbating sand scarcity, ecosystem destruction, and social conflict. We also show that despite industry efforts to reduce CO_2_ emissions by ∼20% per unit of production, mainly through clinker substitution and improved thermal efficiency, increased production has outweighed these gains. Consequently, concrete-related CO_2_ emissions have tripled between 1990 and 2020, and its contribution to global emissions has risen from 5% to 9%. We propose that the policy agenda should focus more on limiting production growth by changing how concrete structures are designed, constructed, used, and disposed of to address the sand and climate crises.

## Highlights

• Global concrete production quadrupled from 1990 to 2020
• Demand for virgin concrete aggregates exceeded the extraction of all fossil fuels
• The concrete industry’s share of global CO_2_ emissions rose to 9% in 2020

## Graphical abstract

**Figure:**
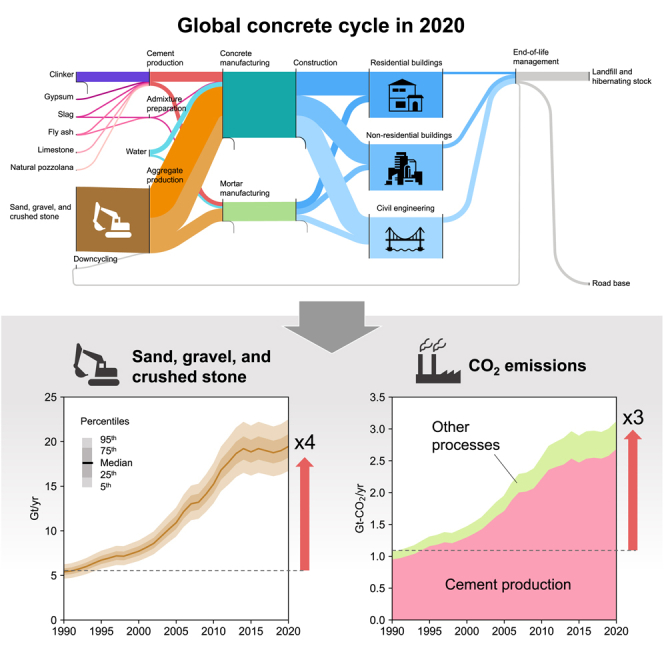

## Introduction

Concrete forms the backbone of modern society, but its massive production poses fundamental and compounding sustainability challenges: climate change and destructive resource extraction.^1^ The underlying cause of these challenges lies in the constituents of concrete. Concrete is composed primarily of cement, (fine and coarse) aggregates, water, and admixtures (where appropriate). The production of cement is one of the most difficult domains to decarbonize due to the inevitable CO_2_ emissions that are induced by carbonate calcination and fuel combustion.^2^ Aggregates (i.e., sand, gravel, and crushed stone), which contribute most to the weight of concrete, are the most extracted materials on the planet by weight.^3^ Recent studies have shown that aggregate extraction, sourcing, and management are largely ungoverned in many regions of the world.^4^^,^^5^ This lack of governance has resulted in far-reaching social and environmental consequences, including ecosystem destruction, illegal mining, and corruption.^6^^,^^7^ These negative impacts, coupled with demand growth beyond what natural resources can support, have fueled concerns about global scarcity, i.e., the sand crisis.^8^^,^^9^

Addressing these challenges requires a systems-based understanding of the material flows and the implications associated with the entire concrete cycle, from resource extraction to processing, manufacturing, construction, use, and end-of-life management. However, despite its universal use in society, the extent to which concrete has been produced, stockpiled, and disposed of is poorly understood on a global scale. Accordingly, information on resource requirements and CO_2_ emissions associated with the global concrete cycle is highly fragmented. Such a lack of systems-based understanding masks the scale of the challenges and the opportunities for future intervention.

Several key studies have addressed some of these knowledge gaps by quantifying the resource requirements and CO_2_ emissions of concrete production.^10^^,^^11^ However, these studies cover only a single year of data and do not reveal long-term trends, including the dynamics of concrete in-use stocks. Some pioneering studies have captured the dynamic changes in concrete production and in-use stocks,^12^^,^^13^^,^^14^^,^^15^ but the associated CO_2_ emissions and their driving forces have not been investigated in detail. A key question is, therefore, to what extent has global concrete production contributed to the extraction of scarce resources and the increase in CO_2_ emissions over time?

This study aims to answer this question by calculating and mapping a series of material flows and CO_2_ emissions associated with the global concrete cycle. The analysis begins with the collection and reconciliation of fragmented statistical data on material flows. The concrete-related CO_2_ emissions are then calculated by compiling a dataset documenting the energy consumption in each process and their associated emission factors. The analysis also reveals the drivers of change in CO_2_ emissions through an index decomposition analysis. Collectively, this study provides a systems-based understanding of the challenges and the opportunities for intervention in the impending sand and climate crises.

## Results

### Increase in concrete production and resource extraction

**Figure 1:**
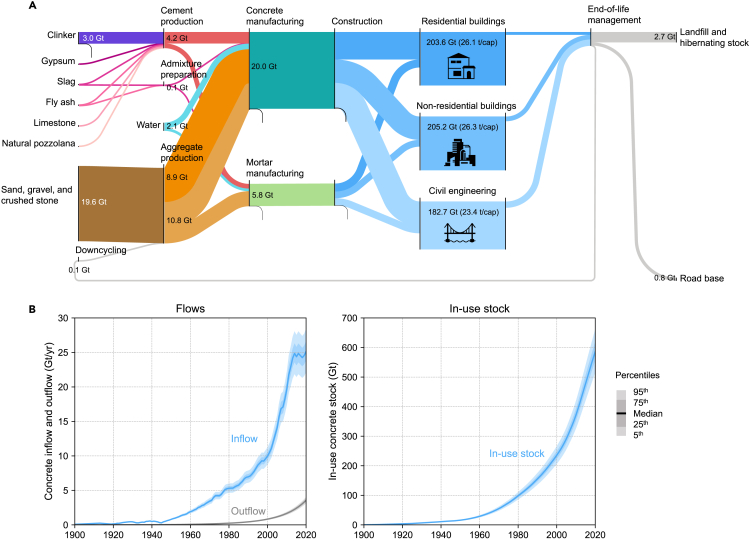
Global map of the concrete cycle (A) Global concrete cycle in 2020. (B) Global inflow, outflow, and in-use stock of concrete, 1900–2020. The Sankey diagram was designed with floWeaver.^17^ Note that cement has additional uses beyond concrete and mortar, such as cementitious solidifiers or cement pastes, but these applications are not included in this study due to the paucity of data.

Based on our analysis, the global production of concrete and mortar (referred to hereafter as concrete for simplicity) quadrupled between 1990 and 2020, reaching ∼26 Gt/yr in 2020 (Figure 1). Accordingly, ∼4 Gt/yr of cement, ∼20 Gt/yr of virgin aggregates, and ∼2 Gt/yr of batch water were used in 2020 to meet the annual concrete demand. The in-use stock of concrete (i.e., concrete that is currently used in buildings and infrastructure) amounted to approximately 590 Gt (76 t per capita) in 2020. It is worth noting that this in-use stock is significantly larger than that of other widely used bulk materials, such as steel, aluminum, paper, and plastic.^16^ Looking further downstream, the mass of end-of-life concrete generated in 2020 (∼4 Gt/yr) was equivalent to only approximately 14% of annual concrete production, most of which was either landfilled, turned into hibernating stock, or downcycled as road-base material. The limited amount of end-of-life concrete, combined with the limited circularity rate, resulted in recycled concrete aggregates accounting for less than 1% of total concrete aggregate production.

**Figure 2:**
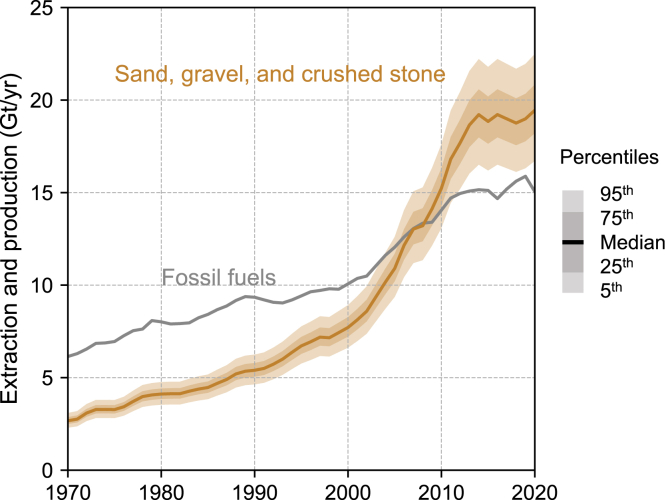
Requirements for virgin concrete aggregates (sand, gravel, and crushed stone) and extraction of all fossil fuels, 1970–2020 Fossil fuel extraction data were obtained from the International Resource Panel’s Global Material Flows Database.^18^

A consequence of such rapid growth in concrete production and limited circularity is a surge in the requirements for virgin aggregates (i.e., sand, gravel, and crushed stone). In 1990, virgin aggregates that were used for concrete manufacturing accounted for only about 60% of all fossil fuel extraction (Figure 2). However, the gap gradually narrowed and finally reversed in the 2000s. Currently, the requirements for virgin concrete aggregates (∼20 Gt/yr) far exceed the extraction of all fossil fuels (∼15 Gt/yr).

Reliable data on total aggregate production are scarce, but global aggregate production in 2020 is estimated to be approximately 42 Mt/yr.^9^ This means that the virgin aggregates used for concrete manufacturing account for approximately half of all aggregate production. Clearly, the increase in concrete production is a major factor underlying the unsustainable use of aggregates, raising scarcity concerns, damaging ecosystems, and fueling social conflict.^3^

### Emissions reduction efforts offset by production growth

**Figure 3:**
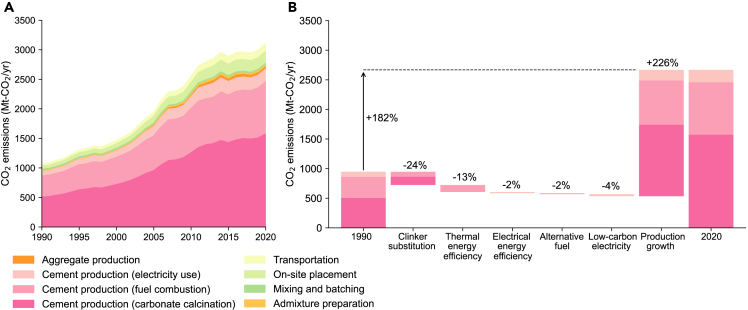
CO_2_ emissions of the global concrete cycle (A) CO_2_ emissions associated with the global concrete cycle for the period 1990–2020. (B) Driving forces underlying the changes in cement-related CO_2_ emissions in 2020 relative to 1990.

The sustainability issues associated with the increase in concrete production are not only restricted to destructive resource extraction but also extend to climate change. According to our analysis, concrete-related global CO_2_ emissions tripled in the last 30 years, reaching ∼3100 Mt-CO_2_/yr in 2020 (Figure 3A). Emissions related to carbonate calcination (i.e., process emissions) account for the largest share (∼51%), followed by fuel combustion (∼29%) and electricity use (∼7%). The next largest contributors toward CO_2_ emissions are on-site placement (∼6%) and transportation (∼4%). The contributions of aggregate production (∼2%), mixing and batching (∼1%), and admixture preparation (<1%) are relatively small, resulting in cement production-related emissions accounting for 86% of the total emissions from the concrete cycle. In contrast, over the same 30-year period, total energy-related CO_2_ emissions (i.e., excluding land-use change) increased by a factor of 1.5.^19^ Consequently, the share of the concrete industry in global energy-related CO_2_ emissions increased from approximately 5% in 1990 to 9% in 2020.

An important question is which factors have contributed the most to the change in CO_2_ emissions? We explored the drivers of changes in CO_2_ emissions associated with cement production using a logarithmic mean Divisia index I approach.^20^ The results show that while the mitigation measures implemented by the cement industry have indeed contributed to emission savings per unit of production, production growth has had the greatest impact, outweighing the impact of these efforts (Figure 3B). Of the various abatement efforts that were implemented by the cement industry between 1990 and 2020, the largest share of emission savings (223 Mt-CO_2_/yr) was attributed to substituting clinker with supplementary cementitious materials (e.g., blast furnace slag and fly ash). Thermal and electrical energy efficiency improvements resulted in further emission savings of 119 Mt-CO_2_/yr and 21 Mt-CO_2_/yr, respectively. Additional emission savings through the use of alternative fuels and electricity decarbonization amounted to 16 Mt-CO_2_/yr and 25 Mt-CO_2_/yr, respectively. Collectively, the abatement efforts of the cement industry resulted in emission savings of 414 Mt-CO_2_/yr in 2020, which is equivalent to 44% of 1990 emissions. In turn, CO_2_ emissions per unit of cement production were reduced by ∼20% between 1990 and 2020. However, the 4-fold increase in cement production has exceeded these savings, resulting in a nearly 3-fold increase in emissions in 2020 compared to 1990 levels. Namely, the increased contribution of the concrete industry to total emissions is primarily due to the fact that the industry’s efforts to reduce CO_2_ emissions have been outweighed by the increase in the societal demand for cement and concrete.

## Discussion

Ultimately, the findings of this study highlight the growing role that the global concrete cycle plays in the impending sand and climate crises. The need for sand, gravel, and crushed stone for concrete manufacturing already far exceeds the extraction of all fossil fuels, exacerbating the global sand crisis. Clearly, how we produce and use concrete holds the key to addressing the issue. It is also important to note that, despite the considerable efforts by the cement and concrete industry to reduce CO_2_ emissions, the CO_2_ savings were entirely outweighed by the surge in concrete production in recent decades. These findings demonstrate the difficulties of tackling resource conservation and affecting deep emissions reductions while continuing to expand cement and concrete production. Improvements in production process efficiency have always been offset by production growth. We propose that the policy agenda should be focused more on how to limit the growth of cement and concrete production by changing the way that concrete structures are designed, constructed, used, and disposed of.

Multiple lines of evidence have shown that those efforts produce significant resource and emission savings throughout the design, construction, use, and end-of-life phases.^21^ For example, in the design phase, performance-based design methods allow architects and contractors to design concrete mixtures that meet the necessary mechanical and durability requirements with less cement.^22^ Post-tensioning techniques can make parts of concrete elements thinner by stressing the rebar in concrete floor slabs before applying external loads.^23^ There is also an important opportunity to almost halve the use of materials in the building frame through better early design, including optimized grid layout and decking choices.^24^ In the construction phase, construction waste can be reduced by promoting prefabrication methods, improving engineering specifications, and using materials that were over-ordered for other purposes.^25^ In the use phase, more intensive use of buildings by living in multi-family homes, sharing office space, or simply by living in smaller homes can reduce the building floor space required to provide the same level of service.^26^^,^^27^ Moreover, extending the useful life of buildings and infrastructure could curb the demand for new reconstruction.^25^ At the end-of-life phase, modularized components could be reused in new construction projects to avoid producing concrete for new products.^28^^,^^29^ The significant resource and emission savings potential from these actions has already been identified.^30^^,^^31^ What is needed is a restructuring of the policy agenda.

Sand, gravel, and crushed stone have been largely ignored in international targets and goals, including the Sustainable Development Goals.^32^ This has led to limited recognition of the importance of efficient use of these materials by relevant stakeholders.^33^^,^^34^ Discussions on climate change mitigation focus heavily on supply-side technologies,^25^ and efficient material use is often off the table.^35^ With the recognition of the role of concrete in both the sand and climate crisis, it is now crucial to establish a policy agenda that promotes its efficient use.

With each new planned building or infrastructure development, we have an opportunity to ask: Do we really need to build more? Can we make do with less? Can we adapt what we already have? Asking such questions could offer a unique opportunity to address both sand and climate crises in one stroke. The findings of this study provide a foundation for discussions in these areas and can inform the development of effective policy solutions.

### Limitations of the study

It is important to note that all of the estimates in this study are based on disparate and incomplete data, which have a high degree of uncertainty. Monte Carlo simulations attempt to capture such uncertainty, but the uncertainty range of system variables is not based on actual measurements. Therefore, the uncertainty range should not be interpreted as an exact range but only as being sufficient to determine whether the trends in the estimation results are sufficiently robust. Simplification of cement applications, such as excluding cementitious solidifiers from the analysis, is another limitation of the study. According to a study conducted in the UK, approximately 4% of cement is used for applications other than concrete and mortar.^23^ Better data collection in these areas is an important future task.

## STAR★Methods

### Key resources table

**Table:** 

REAGENT or RESOURCE	SOURCE	IDENTIFIER
**Deposited data**		

The input data and model results of this study	This study	https://github.com/takumawatari/concrete-flows-global
Cement production	US Geological Survey	https://www.usgs.gov/centers/national-minerals-information-center/cement-statistics-and-information
Cement ingredients	Global Cement and Concrete Association	https://gccassociation.org/sustainability-innovation/gnr-gcca-in-numbers/
Thermal efficiency in the cement kiln	Global Cement and Concrete Association	https://gccassociation.org/sustainability-innovation/gnr-gcca-in-numbers/
Milling/grinding electrical efficiency	Global Cement and Concrete Association	https://gccassociation.org/sustainability-innovation/gnr-gcca-in-numbers/
Carbon intensity of fuel mix	Global Cement and Concrete Association	https://gccassociation.org/sustainability-innovation/gnr-gcca-in-numbers/

**Software and algorithms**		

The model code used for this study	This study	https://github.com/takumawatari/concrete-flows-global

### Resource availability

#### Lead contact

Further information and requests for resources should be directed to, and will be fulfilled by, the lead contact, Takuma Watari (watari.takuma@nies.go.jp).

#### Materials availability

This study did not generate new unique reagents.

### Method details

#### Constructing a global map of the concrete cycle

The map of the global concrete cycle is built on a systems model that tracks the flows and stocks of relevant resources from material production to end-of-life management. The starting point of the model is cement production, for which global data are available (Figure S1). Cement is produced primarily from clinker, an intermediate product in cement production that is obtained by heating a mixture of calcareous and siliceous materials to approximately 1450°C. The produced clinker is then ground with gypsum and additives to form cement. Material flows resulting from cement production are calculated using cement composition data (Figure S2). Cement kiln dust (CKD) to be landfilled is captured by multiplying the estimated clinker production by the CKD generation rate and its landfill rate (Table S3).

The cement produced is used primarily in the production of concrete and mortar. Concrete is a mixture of cement, coarse aggregate, fine aggregate, water, and admixtures, while mortar does not include coarse aggregates. Aggregate, water, and admixture requirements are estimated using data on the share of cement used in concrete, mortar and their mixtures (Figure S3, Tables S1, and S2). Although cement is also used for other applications, such as for cementitious solidifiers or cement pastes, these are not considered in this study due to the paucity of data. The estimated aggregate requirements are then linked to the mining of sand, gravel, and crushed stone. The extraction of virgin aggregates (i.e., sand, gravel, and crushed stone) is quantified as the total aggregate requirement minus recycled aggregates.

Concrete is used primarily as ready-mixed concrete or as precast products for buildings and infrastructure. This study uses global data for the end-use share to classify concrete applications into three categories: residential buildings, non-residential buildings, and civil engineering applications (Figure S4). In-use concrete stocks are estimated using an inflow-driven dynamic material flow analysis (MFA), assuming a specific lifetime for each use category (Figure S5).^15^ This is a time-cohort-type approach that estimates the in-use concrete stock each year based on the total inflow of concrete embedded in the remaining buildings and infrastructure. Specifically, assuming that the flow of concrete into the in-use stock phase in year t is I(t), and the flow of concrete out of the in-use stock phase in year t is O(t), then the in-use stock in year t, S(t), can be defined as follows:

(Equation 1) $$S\left(t\right)=\sum_{t^{\prime }=0}^{t}\left(I\left(t^{\prime }\right)-O\left(t^{\prime }\right)\right)$$

where:

(Equation 2) $$O\left(t\right)=\sum_{t^{\prime }=0}^{t}I\left(t^{\prime }\right)\frac{1}{\sqrt{2\pi \sigma^{2}}}\exp\left(-\frac{{\left(t-t^{\prime }-\mu \right)}^{2}}{2\sigma^{2}}\right)$$

in which, μ is the mean lifetime and σ is the standard deviation of the lifetime distribution.

When the concrete used in buildings and infrastructure reaches the end of its useful life, it is crushed into large pieces. The crushed concrete waste is then either landfilled or sorted and downcycled for use as road-base materials or aggregates in lieu of virgin aggregates sourced from mining. There are also so-called hibernating stocks, which remain *in situ* after they are no longer used or are not removed from the demolition site and are left to mix with the soil and sand on-site (e.g., some foundation piles). These flows are estimated by multiplying the estimated concrete waste by its treatment rate (Figure S6).

The main sources of data are as follows: cement production^12^^,^^36^; cement ingredients^37^; shares of cement used in concrete and mortar^38^; material requirement per unit of cement in concrete production^39^^,^^40^; material requirement per unit of cement in mortar production^39^^,^^40^; market share of concrete end-uses^41^; average lifetime of each end-use^41^ and fate of end-of-life concrete.^12^^,^^14^^,^^42^

The uncertainty of estimates derived using disparate and incomplete data is captured by using Monte Carlo simulations. The uncertain system variables considered are cement production, market share of concrete and mortar, lifetime, concrete mixture, and mortar mixture, with a range of uncertainties based on several literature sources (Tables S1, S2, and S5).^14^^,^^15^^,^^41^^,^^43^ It is important to note that the uncertainty captured here is not based on actual measurements and should not be interpreted as the exact range. Rather, the aim was only to determine whether the observed trends are robust or not.

#### Quantifying CO_2_ emissions of the concrete cycle

CO_2_ emissions associated with the concrete cycle are calculated based on a comprehensive dataset documenting the energy consumption in each process and their associated emission factors (Figures S7–S10 and Table S4). The emission sources considered in this study are broadly classified into cement production, coarse aggregate production, fine aggregate production, admixture preparation, mixing and batching, and transportation activities. Emissions from the use phase are excluded from the model due to difficulties associated with assigning them to a single material.

The main sources of data are as follows: thermal efficiency in the cement kiln^37^; milling/grinding electrical efficiency^37^; carbon intensity of fuel mix^37^; emission factor of electricity generation,^44^^,^^45^ and CO_2_ emission factor for various processes associated with the concrete cycle.^11^^,^^46^^,^^47^^,^^48^

#### Decomposing the drivers of changes in CO_2_ emissions

This study identifies the drivers of changes in carbon emissions associated with cement production using an index decomposition analysis. This is done using a logarithmic mean Divisia index I approach because of its theoretical basis, adaptability, ease of use and interpretation of results, and complete decomposition.^20^ The change in carbon emissions from cement production (ΔC) from the base year $t_{1}$ to the comparison year $t_{2}$ is given by the following equation:

(Equation 3) $$\Delta C=\underset{Process}{\underset{︸}{\Delta C_{act}^{\left(p\right)}+\Delta C_{chi}^{\left(p\right)}}}+\underset{Fuel\;combustion}{\underset{︸}{\Delta C_{act}^{\left(f\right)}+\Delta C_{chi}^{\left(f\right)}+\Delta C_{eff}^{\left(f\right)}+\Delta C_{emi}^{\left(f\right)}}}+\underset{Electricity\;use}{\underset{︸}{\Delta C_{act}^{\left(e\right)}+\Delta C_{eff}^{\left(e\right)}+\Delta C_{emi}^{\left(e\right)}}}$$

where:

(Equation 4) $$\Delta C_{act}^{\left(p\right)}=\frac{C_{t_{2}}^{\left(p\right)}-C_{t_{1}}^{\left(p\right)}}{\ln\;C_{t_{2}}^{\left(p\right)}-\ln\;C_{t_{1}}^{\left(p\right)}}\ln\left(\frac{Q_{cem,t_{2}}}{Q_{cem,t_{1}}}\right)$$

(Equation 5) $$\Delta C_{chi}^{\left(p\right)}=\frac{C_{t_{2}}^{\left(p\right)}-C_{t_{1}}^{\left(p\right)}}{\ln\;C_{t_{2}}^{\left(p\right)}-\ln\;C_{t_{1}}^{\left(p\right)}}\ln\left(\frac{Q_{cli,t_{2}}/Q_{cem,t_{2}}}{Q_{cli,t_{1}}/Q_{cem,t_{1}}}\right)$$

(Equation 6) $$\Delta C_{act}^{\left(f\right)}=\frac{C_{t_{2}}^{\left(f\right)}-C_{t_{1}}^{\left(f\right)}}{\ln\;C_{t_{2}}^{\left(f\right)}-\ln\;C_{t_{1}}^{\left(f\right)}}\ln\left(\frac{Q_{cem,t_{2}}}{Q_{cem,t_{1}}}\right)$$

(Equation 7) $$\Delta C_{chi}^{\left(f\right)}=\frac{C_{t_{2}}^{\left(f\right)}-C_{t_{1}}^{\left(f\right)}}{\ln\;C_{t_{2}}^{\left(f\right)}-\ln\;C_{t_{1}}^{\left(f\right)}}\ln\left(\frac{Q_{cli,t_{2}}/Q_{cem,t_{2}}}{Q_{cli,t_{1}}/Q_{cem,t_{1}}}\right)$$

(Equation 8) $$\Delta C_{\mathrm{eff}}^{\left(f\right)}=\frac{C_{t_{2}}^{\left(f\right)}-C_{t_{1}}^{\left(f\right)}}{\ln\;C_{t_{2}}^{\left(f\right)}-\ln\;C_{t_{1}}^{\left(f\right)}}\ln\left(\frac{E_{t_{2}}^{\left(f\right)}/Q_{cli,t_{2}}}{E_{t_{1}}^{\left(f\right)}/Q_{cli,t_{1}}}\right)$$

(Equation 9) $$\Delta C_{\mathrm{emi}}^{\left(f\right)}=\frac{C_{t_{2}}^{\left(f\right)}-C_{t_{1}}^{\left(f\right)}}{\ln\;C_{t_{2}}^{\left(f\right)}-\ln\;C_{t_{1}}^{\left(f\right)}}\ln\left(\frac{C_{t_{2}}^{\left(f\right)}/E_{t_{2}}^{\left(f\right)}}{C_{t_{1}}^{\left(f\right)}/E_{t_{1}}^{\left(f\right)}}\right)$$

(Equation 10) $$\Delta C_{act}^{\left(e\right)}=\frac{C_{t_{2}}^{\left(e\right)}-C_{t_{1}}^{\left(e\right)}}{\ln\;C_{t_{2}}^{\left(e\right)}-\ln\;C_{t_{1}}^{\left(e\right)}}\ln\left(\frac{Q_{cem,t_{2}}}{Q_{cem,t_{1}}}\right)$$

(Equation 11) $$\Delta C_{\mathrm{eff}}^{\left(e\right)}=\frac{C_{t_{2}}^{\left(e\right)}-C_{t_{1}}^{\left(e\right)}}{\ln\;C_{t_{2}}^{\left(e\right)}-\ln\;C_{t_{1}}^{\left(e\right)}}\ln\left(\frac{E_{t_{2}}^{\left(e\right)}/Q_{cem,t_{2}}}{E_{t_{1}}^{\left(e\right)}/Q_{cem,t_{1}}}\right)$$

(Equation 12) $$\Delta C_{\mathrm{emi}}^{\left(e\right)}=\frac{C_{t_{2}}^{\left(e\right)}-C_{t_{1}}^{\left(e\right)}}{\ln\;C_{t_{2}}^{\left(e\right)}-\ln\;C_{t_{1}}^{\left(e\right)}}\ln\left(\frac{C_{t_{2}}^{\left(e\right)}/E_{t_{2}}^{\left(e\right)}}{C_{t_{1}}^{\left(e\right)}/E_{t_{1}}^{\left(e\right)}}\right)$$

in which the variables and parameters are defined as follows: $\Delta C_{act}$ is the activity effect determined by the cement production ($Q_{cem}$). $\Delta C_{cli}$ is the clinker ratio effect determined by the clinker-to-cement ratio ($Q_{cli}/Q_{cem}$). $\Delta C_{eff}$ is the energy efficiency effect determined by the thermal or electrical energy efficiency ($E/Q_{cli}$ οr $E/Q_{cem}$). $\Delta C_{emi}$ is the emission factor effect determined by the carbon intensity of the fuel mix or electricity grid (C/E). p is the clinker production process. f is the fuel combustion process. e is the electricity use process. Since this study treats the carbon intensity of carbonate calcination as time-invariant, the above equation does not include this factor.

## Data Availability

• The input data and model results of this study have been deposited on GitHub and are publicly available as of the date of publication. DOIs are listed in the key resources table. • All of the original code has been deposited on GitHub and is publicly available as of the date of publication. DOIs are listed in the key resources table. • Any additional information that is required to reanalyse the data reported in this paper is available from the lead contact upon request.
