# Supplementary material for: Growing role of concrete in sand and climate crises
Source: iScience. 2023 Apr 29;26(5):106782. doi: 10.1016/j.isci.2023.106782 (PMC10214720; doi:10.1016/j.isci.2023.106782)
Supplement: Document S1. Figures S1–S10 and Tables S1–S5 [file mmc1.pdf]

iScience, Volume *26*

Supplemental information

Growing role of concrete

in sand and climate crises

Takuma Watari, Zhi Cao, André Cabrera Serrenho, and Jonathan Cullen

Supplementary figures and tables

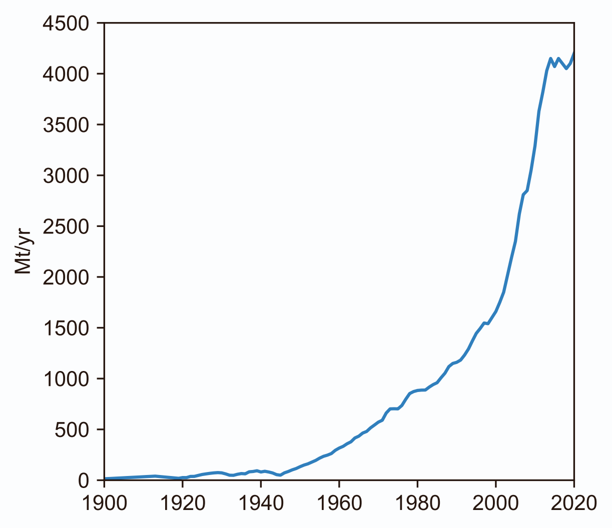

Figure S1 Cement production, 1900-2020.

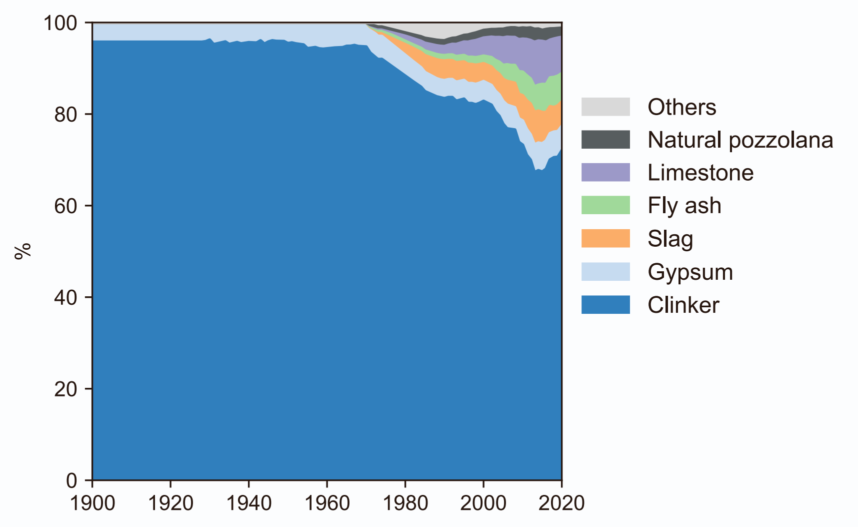

Figure S2 Cement ingredients, 1900-2020.

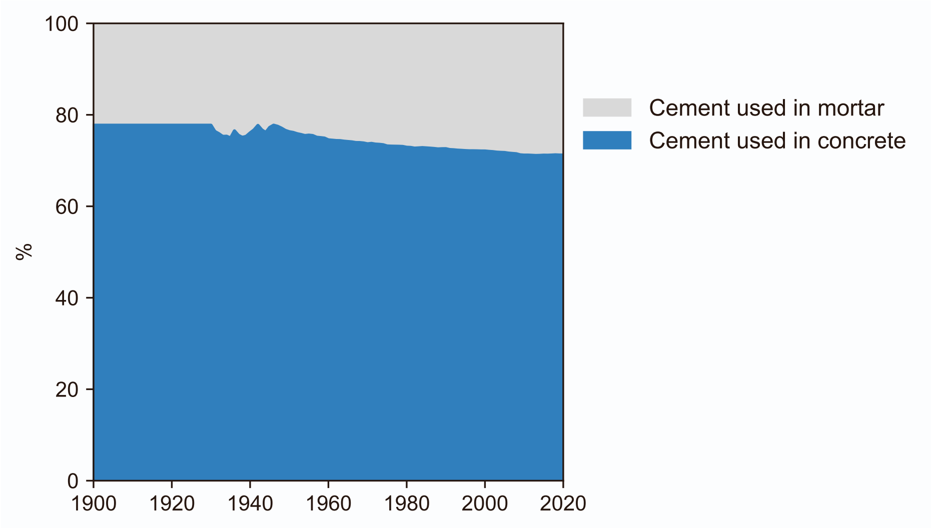

Figure S3 Shares of cement used in concrete and mortar, 1900-2020.

Table S1 Material requirement per unit of cement in concrete production. [Unit: kg-material/kg-cement]

Item	Mean	Min	Max
Water	0.49	0.38	0.60
Fine aggregates	2.26	1.93	2.59
Coarse aggregates	2.92	2.21	3.62
Mineral admixtures	0.01	0.01	0.01

Table S2 Material requirement per unit of cement in mortar production. [Unit: kg-material/kg-cement]

Item	Mean	Min	Max
Water	0.57	0.57	0.57
Fine aggregates	3.18	2.48	3.88
Coarse aggregates	-	-	-
Mineral admixtures	0.01	0.01	0.01

Table S3 Other modeling parameters. [Unit: %]

Item	Value
Manufacturing yield	98.4
Construction yield	98.5
CKD generation rate	6.0
Proportion of landfilled CKD	80.0

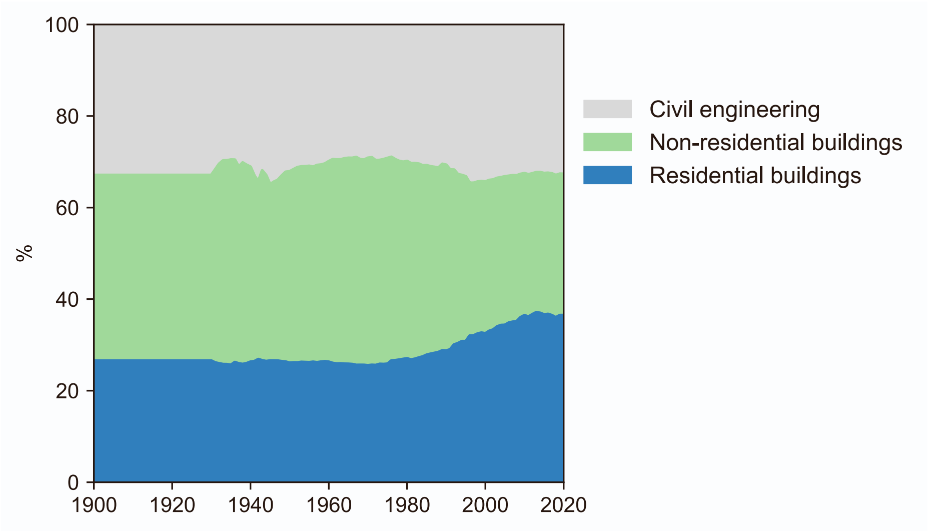

Figure S4 Market share of concrete end-uses, 1900-2020.

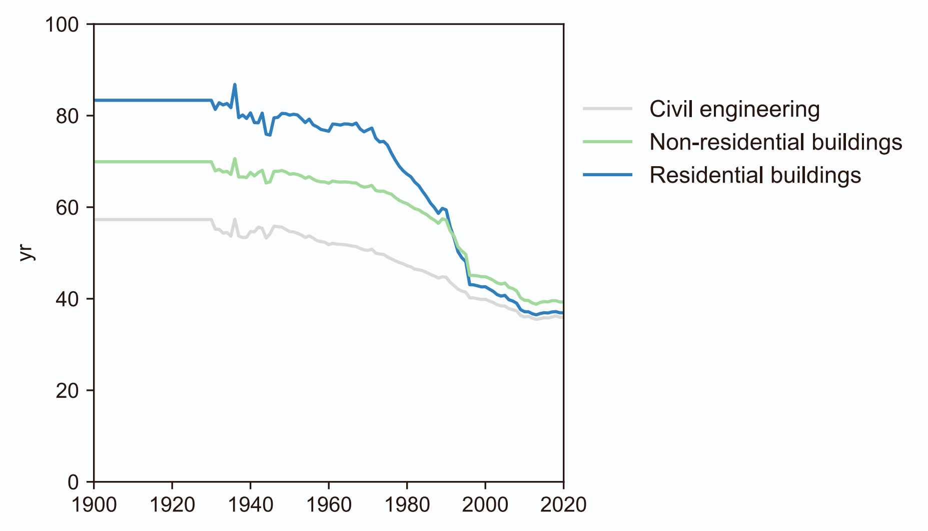

Figure S5 Average lifetime of each end-use, 1900-2020.

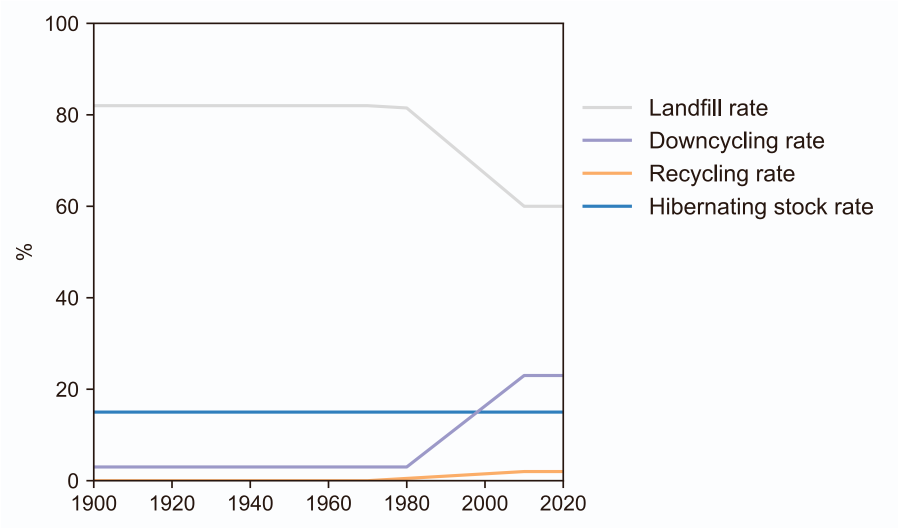

Figure S6 Fate of end-of-life concrete, 1900-2020.

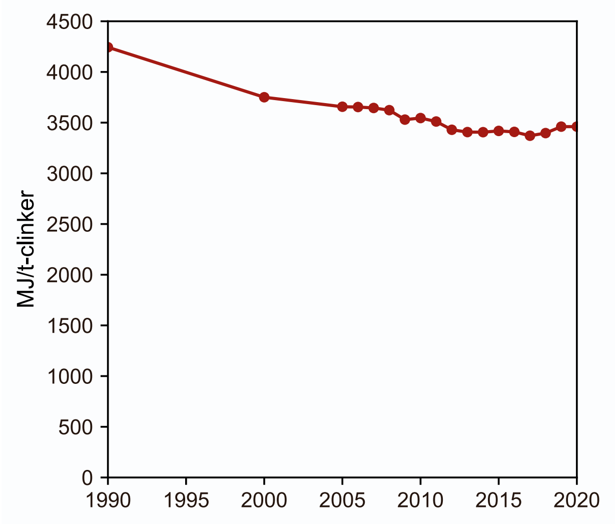

Figure S7 Thermal efficiency in the cement kiln, 1990-2020.

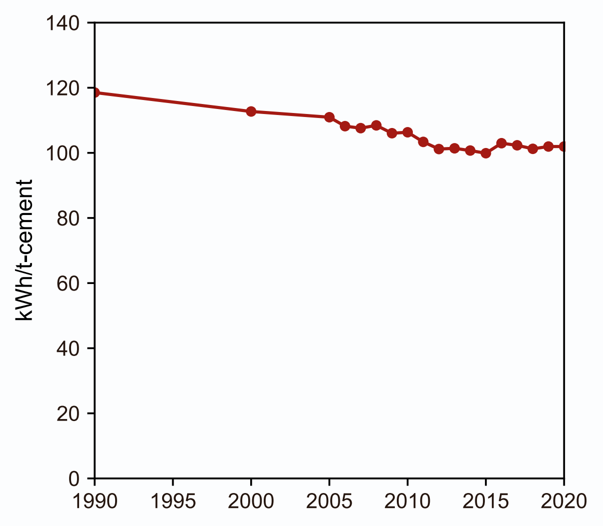

Figure S8 Milling/grinding electrical efficiency, 1990-2020.

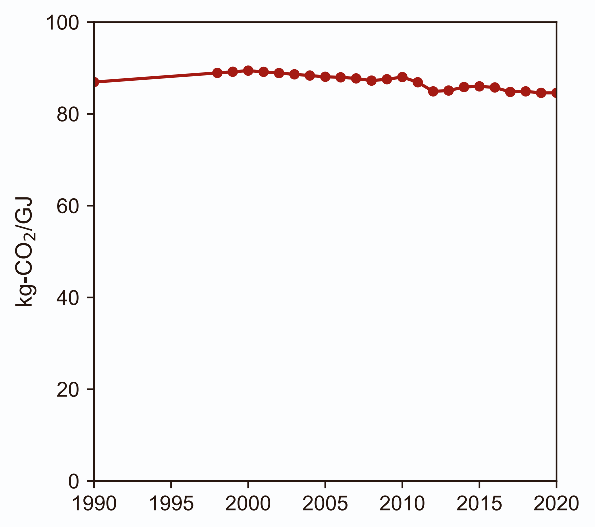

Figure S9 Carbon intensity of fuel mix, 1990-2020.

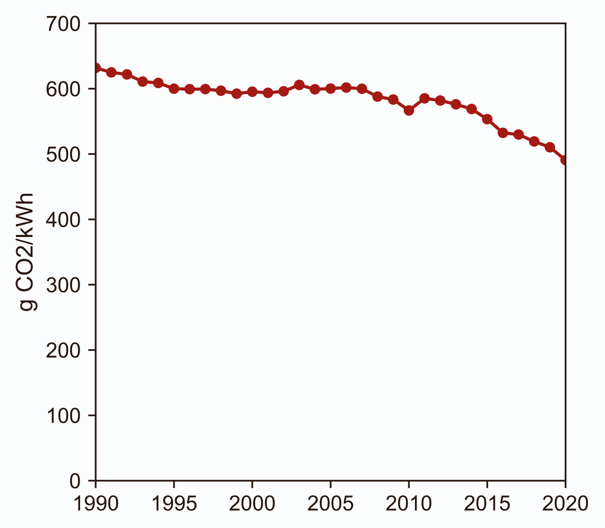

Figure S10 Emission factor of electricity generation, 1990-2020.

Table S4 CO_2 emission factor for various processes associated with the concrete cycle.

Item	Value	Unit
Emission factor for virgin aggregate production	3.2	kg-CO_2/t
Emission factor for recycled aggregate production	3.2	kg-CO_2/t
Emission factor for mixing and batching	1.2	kg-CO_2/t
Emission factor for on-site placement	7.8	kg-CO_2/t
Emission factor for cement transportation	8.9	kg-CO_2/t
Emission factor for admixture transportation	2.4	kg-CO_2/t
Emission factor for virgin aggregate transportation	4.4	kg-CO_2/t
Emission factor for buried aggregate transportation	4.4	kg-CO_2/t
Electricity use for slag preparation	363.9	kWh/t
Electricity use for fly ash preparation	119.4	kWh/t

Table S5 Uncertainty ranges for Monte Carlo simulations. The uncertainty range is expressed as relative variation of the respective parameter (in %) and contain one standard deviation.

Item	Distribution	Uncertainty range
Cement production	Normal	±5%
Market share of concrete and mortar cement	Normal	±5%
Lifetime	Normal	±10%
Concrete mixture	Uniform	Table S1
Mortar mixture	Uniform	Table S2
